# Targeting Autophagy for Otoprotection: Translating Basic Mechanisms into Clinical Strategies

**DOI:** 10.3390/ijms27052229

**Published:** 2026-02-27

**Authors:** Fei Wang, Tiantian Zhang, Bin Bai, Lian Hui, Yan Wang, Jian Zang

**Affiliations:** 1 Department of Otolaryngology, The First Hospital of China Medical University, Shenyang 110001, China; feiwang@cmu.edu.cn (F.W.); lhui@cmu.edu.cn (L.H.); 2 Department of Geriatrics, The First Hospital of China Medical University, Shenyang 110001, China; ttzhang@cmu.edu.cn; 3 Department of Dermatology, The First Hospital of China Medical University, Key Laboratory of Immunodermatology, Ministry of Education and NHC, National Joint Engineering Research Center for Theranostics of Immunological Skin Diseases, Shenyang 110001, China; baibin0517@163.com

**Keywords:** autophagy, sensorineural hearing loss, mitophagy, pexophagy, mTOR signaling, therapeutic targets

## Abstract

Sensorineural hearing loss (SNHL), the predominant form of global hearing impairment, stems from the irreversible loss of inner ear sensory cells and neurons. Since mammalian cochlea lacks regenerative capacity, cell death represents a final common pathway for diverse insults. Current therapies are merely compensatory, underscoring an urgent need for mechanistic, targeted interventions. Autophagy, a critical homeostatic process, plays complex and dynamic roles in the cochleae. This review synthesizes current evidence on its regulation, highlighting its stage-specific and dual roles in SNHL. We emphasize mitophagy and its context-dependent effects on cell survival. Critically, we discuss an emerging therapeutic paradigm: a dual-phase autophagy modulation strategy. This approach proposes enhancing cytoprotective autophagy in early stages to maintain homeostasis, while inhibiting excessive autophagic flux later to prevent catastrophic cell death. This precision-targeting framework holds significant promise for guiding novel drug development and future clinical translation, moving beyond symptomatic management towards transformative treatment.

## 1. Introduction

Sensorineural hearing loss (SNHL) represents a predominant form of permanent auditory impairment, affecting millions worldwide and posing a significant burden on quality of life, social interaction, and cognitive function [1]. The core pathology of SNHL lies in the irreversible damage or loss of critical sensory cells, the cochlear hair cells (HCs) and their associated spiral ganglion neurons (SGNs) [2,3]. As terminally differentiated cells with minimal regenerative capacity in mammals, the demise of HCs and SGNs is a final common pathway in hearing loss induced by aging, noise overexposure, ototoxic drugs, and genetic mutations [4]. Despite SNHL’s high prevalence, treatments are critically limited. Current interventions like hearing aids and cochlear implants offer support but fail to restore natural hearing or halt progression. This stark disparity between the scale of the problem and the inadequacy of available treatments underscores an urgent, unmet clinical need for mechanistically grounded, targeted therapies that can protect or rescue auditory cells. In this context, the cellular self-degradation process of autophagy has emerged as a pivotal and druggable pathway, offering a promising breakthrough for innovative therapeutic strategies aimed at preserving auditory function by targeting the very roots of cochlear cell death.

Cellular homeostasis in the highly metabolically active and stress-prone cochlear environment is maintained by crucial quality control pathways, among which autophagy plays a central role [5]. Autophagy, particularly macroautophagy, is an evolutionarily conserved intracellular degradation process essential for clearing damaged organelles, misfolded proteins, and invasive pathogens [6]. This multi-step process, orchestrated by a suite of autophagy-related gene (ATG) proteins, unfolds sequentially: it begins with initiation and phagophore nucleation, proceeds through elongation and encapsulation of cargo to form the double-membraned autophagosome, and culminates in fusion with lysosomes for cargo degradation and recycling within the autophagolysosome [7,8]. Under physiological conditions, basal autophagy serves as a pro-survival mechanism, critical for maintaining the health and longevity of post-mitotic HCs and SGNs by, for instance, mitigating oxidative stress through selective mitophagy [4,9,10,11].

Autophagy in SNHL acts as a context-dependent double-edged sword, where its dysregulation leads to divergent outcomes: insufficient flux promotes toxic accumulation in age-related hearing loss (ARHL) and genetic hearing loss, while excessive activation exacerbates noise-induced or ototoxic damage [12,13]. For this narrative review, we conducted a comprehensive literature search using the PubMed, Web of Science, and Scopus databases, covering publications up to December 2025. The search terms included combinations of ‘autophagy,’ ‘mitophagy,’ ‘pexophagy,’ ‘sensorineural hearing loss,’ ‘hair cells,’ ‘spiral ganglion neurons,’ ‘ototoxicity,’ ‘age-related hearing loss,’ and ‘noise-induced hearing loss.’ This review addresses the critical knowledge gap of stage-specific autophagic alterations by systematically examining dysregulation across initiation, elongation, and degradation stages in major SNHL subtypes [14,15]. Our objective is to synthesize aberrant molecular targets and map disease–stage–target relationships, thereby providing a refined mechanistic framework to illuminate novel therapeutic avenues for precise inner ear cytoprotection.

## 2. Molecular Mechanisms of Autophagy in SNHL

The autophagy process comprises four key stages: (1) Initiation and phagophore formation: Cellular stresses inhibit mammalian target of rapamycin complex 1 (mTORC1) or activate AMP-activated protein kinase (AMPK), leading to the phosphorylation and activation of the Unc-51 like autophagy activating kinase 1 (ULK1) complex. This initiates phagophore formation, a process involving proteins like Beclin-1 (BECN1) and Vacuolar protein sorting 34 (Vps34) [16]. (2) Autophagosome formation: The phagophore expands via two ubiquitin-like conjugation systems: the ATG12-ATG5-ATG16L1 complex facilitates membrane elongation, while microtubule-associated protein 1A/1B-light chain 3 (LC3) is lipidated to form LC3-II, enabling cargo recognition and autophagosome sealing. (3) Autolysosome formation: The completed autophagosome fuses with a lysosome. (4) Degradation and recycling: The encapsulated cargo is degraded by lysosomal enzymes, and the resulting macromolecules are released back into the cytoplasm for reuse [17]. Given SNHL’s irreversible pathology and current devices’ limited compensatory functions, a decisive shift toward proactive intervention is unavoidable. Delineating stage-specific autophagy dysregulation across SNHL subtypes is paramount, as it reveals a crucial therapeutic window preceding irreversible damage for clinical translation.

**Figure 1:**
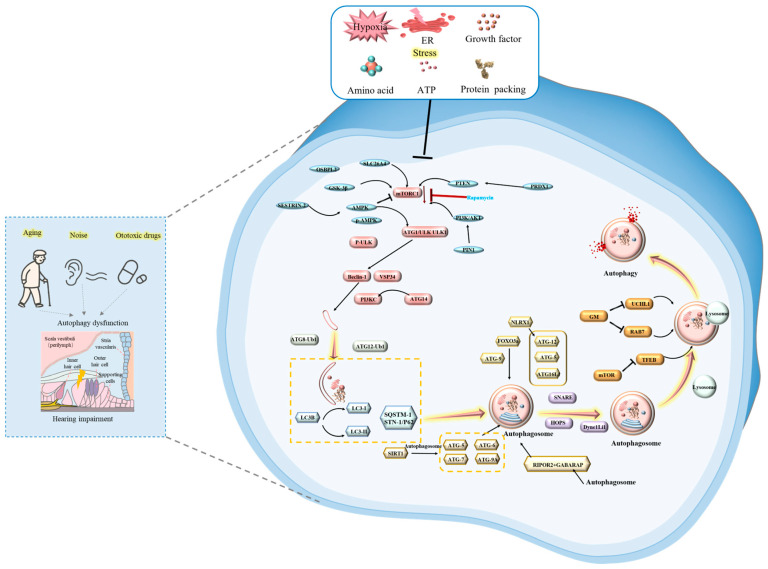
Stage-specific regulatory network of stress-induced autophagy in cochlear cells. Under ototoxic/aging stress, autophagy in cochlear cells proceeds through four stages: (1) Initiation: Stress signals inhibit mTORC1 and activate AMPK, converging on ULK1 to activate the BECN1/PI3KC3 complex for phagophore formation. (2) Elongation: The ATG12-5-16L1 and LC3 systems drive autophagosome formation, regulated by SIRT1, FOXO3a/FOXG1, and NLRX1. (3) Fusion: Autophagosomes are transported (via Dync1li1) and fuse with lysosomes (regulated by Rab7 and UCHL1). (4) Degradation & Recycling: Lysosomal degradation is orchestrated by TFEB; its dysfunction (e.g., in aging or SLC7A14 defects) impairs flux. Key therapeutic targets (e.g., rapamycin, metformin) are indicated.

In subsequent sections, we comprehensively summarize autophagy’s roles in SNHL, with particular emphasis on these four critical stages. Figure 1 shows a schematic diagram of the main molecular mechanisms and regulatory networks in different stages of autophagy regulation in inner ear tissues under stress conditions.

### 2.1. Autophagy Initiation and Regulation

Autophagy initiation is critically governed by the ULK1 complex, whose activity is centrally regulated by the nutrient-sensing kinase mTORC1. Under nutrient-replete conditions, mTORC1 phosphorylates ULK1 at Ser757, suppressing its kinase activity and inhibiting autophagy. Cellular stresses such as nutrient deprivation, energy depletion, hypoxia, and ER stress relieve this inhibition, enabling ULK1 activation. Activated ULK1 then phosphorylatesBECN1, facilitating assembly of the phosphatidylinositol 3-kinase complex (PI3KC: containing BECN1, Vps34, Vacuolar protein sorting-associated protein 15 (Vps15), and ATG14) to drive phagophore formation [16,18,19,20,21]. The translational significance of this pathway is demonstrated by rapamycin, an mTOR inhibitor that enhances autophagic flux in cochlear HCs and SGNs, protecting against ototoxicity induced by cisplatin, gentamicin (GM), neomycin, acoustic trauma, and ARHL in mice [2,9,22,23]. AMPK complements mTORC1 as an energy-sensing regulator of autophagy initiation. It promotes autophagy both indirectly by inhibiting mTORC1 and directly by phosphorylating ULK1, fine-tuning activity to stimuli [24,25,26,27,28]. AMPK dysfunction impairs autophagy and promotes senescence in auditory House Ear Institute-Organ of Corti 1 (HEI-OC1) cells, vital for cell viability. Upstream, Sestrin-2(SESN2) modulates mTORC1 via GATOR2 during amino acid starvation, forming a SESN2/AMPK/mTOR network for stress-induced autophagy [29,30]. SESN2 levels decline in aged and ototoxin-exposed cochleae, and its deletion increases hair cell susceptibility [31,32,33]. MicroRNAs like miR-130b-3p also regulate autophagy; its overexpression suppresses autophagy-related gene 5 (ATG5), Becin-1, and LC3B-II/I, impairing autophagy and contributing to ARHL in models [34]. Other pathways also converge on autophagy initiation. Glycogen synthase kinase-3β (GSK-3β) is a versatile protein kinase that plays roles in various physiological processes and the development of diseases [35]. GSK-3β generally promotes autophagy via mTORC1 inhibition [35,36]. In cisplatin-induced ototoxicity, protein kinase B (AKT) negatively regulates GSK-3β through Ser9 phosphorylation, thereby enhancing autophagy and reducing HCs damage. Interestingly, in noise-induced hearing loss (NIHL), noise exposure triggers reactive oxygen species (ROS) overproduction and activates the AMPK/ULK1 pathway, while downregulating phosphatidylinositol 3-kinase (PI3K) and AKT expression to further facilitate autophagic induction [37,38,39].

Autophagy also plays a critical role in hereditary hearing loss. In Pendred syndrome, caused by mutations in the Solute Carrier Family 26, Member 4 (SLC26A4) gene, the misfolded pendrin protein accumulates in cochlear outer sulcus cells (OSCs), leading to cellular dysfunction [40,41,42]. This accumulation can be mitigated by rapamycin-induced autophagy, which clears the aberrant protein, even at low doses [17,43]. Similarly, mutations in Oxysterol Binding Protein-Like 2 (OSBPL2) inhibit autophagy via dysregulated mTORC1 signaling, resulting in hearing loss that is also amenable to rapamycin treatment [44].

In addition to dealing with genetic defects by clearing mutant proteins, autophagy also plays a key protective role in resisting stress caused by external damage (such as ototoxic drugs). Peroxiredoxin 1 (PRDX1), an antioxidant enzyme highly expressed in cochlear HCs, the lateral wall, and SGNs, promotes autophagy initiation under ototoxic conditions [45,46]. Demonstrated that PRDX1 protects SGNs from cisplatin-induced damage by enhancing autophagic flux. Mechanistically, PRDX1 interacts with phosphatase and tensin homolog (PTEN), enhancing its phosphatase activity to reduce PIP3 levels, thereby suppressing AKT-mTOR signaling and relieving mTOR-mediated inhibition of the ULK1 complex—the core initiator of autophagy [47,48,49]. At the translational regulation level, the RNA-binding protein YT521-B Homology N6-Methyladenosine RNA Binding Protein (YTHDF1) facilitates autophagy initiation by enhancing the translation of autophagy-related gene 14 (ATG14), an essential component of PI3KC required for phagophore nucleation. Overexpression of YTHDF1 elevates ATG14 expression, thereby promoting PI3KC assembly and increasing autophagosome biogenesis, which protects HCs from cisplatin injury [50]. Furthermore, transcriptional regulation plays a vital role in autophagy initiation. Forkhead box O3a (FOXO3a) acts as a master transcriptional activator of multiple core autophagy genes. Under stress, metformin induces autophagy via the AMPK/FOXO3a axis, alleviating cisplatin-induced ototoxicity across models. FOXO3 activation often facilitated by mTORC1 inhibition directly upregulates the expression of initiation-related ATGs, thereby enhancing autophagic capacity and representing a promising target for hearing protection [51,52,53,54,55].

The initiation phase, governed by nutrient-sensing pathways and stress-responsive signals, sets the autophagy process in motion. However, the successful execution of autophagy and its ultimate impact on cell fate hinge upon the subsequent stages where the initial phagophore matures into a functional degradative organelle. Having established how autophagy is triggered, we now turn to the critical molecular events that govern autophagosome maturation and fusion, a process equally vital for maintaining auditory cellular homeostasis.

### 2.2. Autophagosome Maturation and Fusion

Following initiation, autophagosome maturation and fusion are critically regulated by ubiquitin-like conjugation systems and key protein modifiers. The process relies on two core ubiquitin-like (Ubl) systems: the autophagy related 12 (ATG12)–ATG5–autophagy related 16-like 1 (ATG16L1) complex, which facilitates phagophore expansion, and the autophagy related 8 (ATG8) system, involving LC3 and GABA type A receptor-associated protein (GABARAP). LC3 is first cleaved by ATG4B to form LC3-I, which is then conjugated to phosphatidylethanolamine by ATG7 (E1) and ATG3 (E2) enzymes—a step essential for membrane anchoring and autophagosome closure. The autophagy receptor p62/Sequestosome-1 (SQSTM1) recognizes ubiquitinated cargo and recruits it to the growing phagophore, enabling selective degradation [56]. The conversion of LC3-I to LC3-II, coupled with p62/SQSTM1 degradation, serves as a well-established marker of autophagic flux [57]. In addition, autophagy related 9 (ATG9), the only transmembrane autophagy-related protein, contributes to membrane sourcing and transport during autophagosome formation [58].

Beyond these core machinery components, post-translational modifications such as acetylation fine-tune autophagosome maturation. Sirtuins constitute a conserved family of nicotinamide adenine dinucleotide (NAD^+^)-dependent class III histone deacetylases, which are associated with lifespan extension and regulate various physiological processes including deoxyribonucleic acid (DNA) repair, apoptosis, and antioxidant activities [59,60]. Among them, Sirtuin-1 (Sirt1) is the most conserved mammalian ortholog and plays vital roles in cellular homeostasis. In the context of autophagy, Sirt1 promotes autophagosome maturation and fusion by directly deacetylating core autophagy proteins such as ATG5, ATG9, and autophagy protein autophagy related 9A (ATG9A), thereby enhancing autophagic flux under stress conditions [61]. In the auditory system, Sirt1 expression decreases in aged C57BL/6 mice, correlating with reduced autophagy and HCs loss. Correspondingly, Sirt1 knockdown in HEI-OC1 cells leads to impaired autophagosome formation, evidenced by decreased LC3-II and accumulated p62. Importantly, Sirt1 activation protects against both age-related and drug-induced ototoxicity: Xiong et al. reported its cochlear expression in ARHL models, while Pang et al. demonstrated that Sirt1-induced autophagy attenuates cisplatin-induced HC death in mice and zebrafish, specifically through deacetylation of ATG9A to regulate autophagosome biogenesis [62,63,64,65,66].

Beyond Sirt1, the study also identified other key regulators of autophagosome maturation, whose roles may be more complex. Nucleotide-binding domain and leucine-rich repeat-containing receptors family member X1 (NLRX1) represents another key regulator of autophagosome formation, though its role is context-dependent. In HEI-OC1 cells, NLRX1 overexpression amplifies mitochondrial ROS production and activates the Reactive oxygen species/c-Jun N-terminal kinase (ROS/JNK) pathway, exacerbating cisplatin-induced ototoxicity and triggering autophagosome accumulation. Conversely, NLRX1 silencing reduces autophagy activation and enhances cell viability. Mechanistically, NLRX1 promotes assembly of the ATG12-ATG5-ATG16L1 complex, essential for phagophore elongation and autophagosome formation [67,68,69]. The significance of proper autophagosome maturation is further highlighted by findings that ATG16L1 deficiency, due to WD domain loss, causes hearing loss and cochlear defects in mice, underscoring the importance of non-classical autophagy pathways in inner ear homeostasis [70].

Autophagy regulation extends beyond classical complex assembly, intersecting with structural proteins critical for auditory system integrity. A prime example is Rho-family interacting cell polarization regulator 2 (RIPOR2), which is essential for stereocilia morphogenesis and highly expressed in cochlear hair cells (HCs) [71]. Recent studies have uncovered its pivotal role in autophagy activation. Specifically, GABARAP—a known autophagy/mitophagy effector in aminoglycoside-induced ototoxicity—interacts with RIPOR2. Using HEI-OC1 cells and mouse cochlear explants, Li et al. demonstrated that aminoglycosides bind directly to RIPOR2, triggering its rapid translocation from stereocilia to the pericuticular area [72]. This translocation promotes RIPOR2-GABARAP interaction and subsequent autophagy activation. Importantly, unlike the protective autophagy induced by rapamycin, RIPOR2-mediated autophagy in this context is clearly detrimental: knockdown of either RIPOR2 or GABARAP completely prevents hair cell death and hearing loss. This stark contrast underscores that autophagy outcomes depend on the trigger, intensity, and cellular context. Thus, mechanism-based intervention is essential—autophagy should be inhibited when overactivated by structural protein mislocalization (e.g., RIPOR2-driven damage), but enhanced when deficient due to metabolic or oxidative stress (e.g., cisplatin or noise injury). Future studies validating the evolutionary conservation of the RIPOR2-GABARAP interaction will further support their potential as therapeutic targets for aminoglycoside-induced ototoxicity.

The precise regulation of autophagy extends beyond direct protein interactions to include transcriptional levels within the nucleus. In this process, the transcription factor Forkhead Box G1 (FOXG1) plays a pivotal role. FOXG1 is a pivotal transcription factor essential for cochlear development, whose dysfunction impairs HCs formation and neural innervation [73,74]. Beyond its developmental role, FOXG1 critically promotes HC survival under stress by activating autophagy. In inflammatory settings such as lipopolysaccharide (LPS) exposure, FOXG1 upregulation enhances autophagic activity, mitigating oxidative stress and preserving aged HCs viability [11,75]. Similarly, under ototoxic conditions, FOXG1 activation—whether by pharmacological agents such as aspirin or through endogenous regulation—attenuates HCs damage by sustaining autophagic flux. Recent evidence further indicates that FOXG1 ameliorates cisplatin-induced ototoxicity through the regulation of autophagy-related microRNAs, including miR-34a, miR-96, miR-182, and miR-183 [76]. Collectively, these findings establish FOXG1 as a central regulator of autophagy in age-related and ototoxic hearing loss, though its role in other forms of SNHL remains to be fully elucidated [77,78,79].

Regulation of autophagosome maturation and fusion is essential for initiating cellular cargo degradation. However, autophagy success hinges on efficient autophagosome-lysosome fusion to form functional autolysosomes, where degradation and nutrient recycling occur. This fusion step, governed by specific molecular, represents a critical regulatory node; its dysfunction carries profound implications for cochlear cell survival in SNHL.

### 2.3. Autolysosome Formation

Autophagosome-lysosome fusion into autolysosomes is mediated by soluble NSF attachment protein receptor (SNARE) and homotypic fusion and vacuole sorting (HOPS) complexes [80]. Ubiquitin carboxyl-terminal hydrolase isozyme L1 (UCHL1) deficiencies are linked to autophagy pathway impairments during injury or pathology [81]. UCHL1, a deubiquitinating enzyme (DUB) enzyme, stabilizes mono-ubiquitin by cleaving polyubiquitin chains, maintaining the free ubiquitin pool essential for ubiquitin-proteasome system (UPS). Highly expressed in the brain and neuroendocrine system, UCHL1 sustains synaptic structure, stabilizes ubiquitin, and regulates degradation pathways [82]. Kim et al. found GM exposure downregulated UCHL1 in SGNs, lateral wall, and efferent nerves throughout the auditory system [83]. In cochlear cultures and HEI-OC1 cells, UCHL1 deficiency accelerated GM-induced ototoxicity, reducing SGNs and neural fibers. GM also caused time-dependent cochlear UCHL1 reduction. Silencing UCHL1 blocked autophagic flux and inhibited Lysosome-Associated Membrane Glycoprotein 1 (LAMP1)-LC3 colocalization. UCHL1-deficient cells exhibited increased autophagosome accumulation with decreased lysosomal fusion, suggesting GM-induced UCHL1 downregulation may impede autophagosome-lysosome fusion.

The successful delivery of autophagosomes to perinuclear lysosomal compartments depends on intact microtubule-dependent transport, a vital retrograde process primarily driven by the cytoplasmic dynein motor complex. As a critical subunit of cytoplasmic dynein 1, Dynein cytoplasmic 1 light intermediate chain 1 (Dync1li1) plays an indispensable role in transporting autophagosomes toward lysosomes across diverse tissues [84]. Zhang et al. reported high expression of Dync1li1 in cochlear HCs [85]. Notably, genetic knockout or knockdown of Dync1li1 induced pronounced autophagosome accumulation in HCs and HEI-OC1 cells, accompanied by reduced autolysosome formation and elevated levels of LC3B. These findings clearly indicate that loss of Dync1li1 disrupts autophagosome transport to lysosomes, leading to impaired clearance, buildup of autophagosomes, subsequent HCs apoptosis, and hearing loss. Thus, Dync1li1 serves a crucial role in HCs survival by regulating autophagosome trafficking.

Following microtubule-mediated transport, the small GTPase Ras-related protein Rab-7 (Rab7) orchestrates the final fusion step between autophagosomes and lysosomes. In HEI-OC1 cells, GM treatment induced a time-dependent increase in LC3-II, coupled with decreased Rab7 expression and enhanced cell death. As a central regulator of autophagosome–lysosome fusion, Rab7 is essential for maintaining autophagic flux [86]. Beyond fusion, Rab7 also regulates phagosome trafficking and promotes mitophagosome formation, underscoring its necessity in the autophagic pathway [87]. Importantly, rapamycin treatment in GM-exposed cells elevated both Rab7 and cathepsin D levels, resulting in significantly improved cell survival. Following the successful fusion of autophagosomes and lysosomes, the effective degradation of their contents and the integrity of lysosomal function become the next key link that determines the success or failure of autophagy and the fate of cells.

### 2.4. Autophagosome–Lysosome Fusion and Degradation

While the successful fusion of autophagosomes with lysosomes signifies autolysosome formation, the subsequent degradation of cargo and the maintenance of lysosomal functionality are equally pivotal for completing the autophagic process and determining cellular fate in SNHL. As acidic organelles, lysosomes play a critical role in degrading macromolecules into smaller components, facilitating nutrient recycling and cellular salvage [88]. Consequently, lysosomal dysfunction impairs autophagic flux, triggering a cascade of pathological events that ultimately promote apoptotic cell death [89].

Transcription factor EB (TFEB), a key MiT/TFE family member and master regulator of autophagy–lysosomal pathways, controls lysosomal biogenesis and autophagy by activating genes like microtubule-associated proteins 1A/1B-light chain 3B (MAP1LC3B/LC3B) and ATG9B [90]. Phosphorylated TFEB binds 14-3-3 proteins in the cytoplasm under nutrient-rich conditions. Cellular stress triggers TFEB dephosphorylation and nuclear translocation, enhancing lysosomal function and autophagic flux [90]. Compelling evidence underscores TFEB’s critical function in preserving hearing: for instance, mTORC1 inhibition and subsequent TFEB activation via atorvastatin effectively prevents ARHL [91]; additionally, the RONIN-host cytokine C1 (HCF1/HCFC1) complex modulates TFEB activity, thereby reducing hair cell aging [92]; furthermore, Hypoxia-Inducible Factor 1-αlpha (HIF-1α) stabilization and TFEB nuclear localization synergistically shield hair cells against hypoxic damage [93,94]. Conversely, impaired TFEB activation causes lysosomal dysfunction and auditory pathology: sodium arsenite induces TFEB translocation but fails to restore autophagy, worsening damage [95]; disrupted TFEB nuclear translocation occurs in SNHL [96,97,98,99,100], linked to SGNs degeneration and lipofuscin accumulation [101]. mTOR inhibition promotes TFEB translocation, restores autophagy, and protects SGNs, highlighting the mTOR-TFEB axis’s therapeutic potential [101,102,103]. Circadian clock genes directly interact with the autophagy mechanism. CLOCK can acetylate TFEB, regulating its nuclear translocation and subsequent lysosomal biogenesis. These findings suggest that core clock genes control autophagy, at least in part, through the mTOR-TFEB axis, providing a molecular basis for circadian-based interventions. Modulating sleep cycles or targeting these genes may present novel prophylactic strategies for ARHL or NIHL.

Beyond TFEB’s global transcriptional regulation of lysosomal function, the structural integrity and functional homeostasis of lysosomes are equally vital for the completion of autophagy. Their disruption—whether pharmacological or genetic—is a critical factor in the pathogenesis of SNHL. For example, Zhao et al. showed acetaminophen ototoxicity causes lysosomal membrane permeabilization, disrupting autophagosomal degradation in HEI-OC1 cells and cochlear HCs, impairing autophagic flux; N-acetylcysteine (NAC) partially rescues this, indicating oxidative stress contributes [104]. Genetic defects also impair autophagy. Patients with pathogenic Adenosine Triphosphatase (ATPase) H^+^ Transporting, Vacuolar, 1 B-subunit, 1 (ATP6V1B1) mutations often have early-onset SNHL. Zebrafish studies show Atp6v1ba loss causes lysosomal pH imbalance and autophagy dysfunction in inner ear HCs [105]. Similarly, mutations in Solute carrier family 7 (cationic amino acid transporter, y^+^ system, member 14) (SLC7A14), a lysosomal cationic amino acid transporter highly expressed in inner HCs, cause autosomal recessive hearing loss [106]. SLC7A14 mutations serve as a key counterexample, demonstrating that autophagy is overactivated rather than deficient. SLC7A14 encodes a lysosomal cationic amino acid transporter; its dysfunction induces lysosomal stress and results in aberrantly elevated autophagic flux. This excessive autophagy drives HC and photoreceptor loss by depleting essential organelles and promoting autophagic cell death [106]. Consequently, in this genetic context, autophagy inhibition—not activation—represents the appropriate therapeutic strategy. This aligns perfectly with our dual-phase modulation concept: when autophagic flux exceeds a protective threshold due to specific genetic or environmental triggers, suppression becomes necessary to prevent self-digestion and cell death.

These examples collectively underscore that lysosomal dysfunction—whether induced by ototoxic drugs or genetic mutations—can severely disrupt autophagic flux and promote cochlear cell death, highlighting the lysosome as a critical hub in SNHL pathogenesis.

Table 1 summarizes the multiple stages of autophagy and their roles in the pathogenesis of SNHL. While general autophagy is critical for cellular homeostasis, selective autophagy types—such as mitophagy and pexophagy—play specialized roles in protecting auditory cells against specific stressors.

## 3. Selective Autophagy Pathways in Auditory Cell Survival

### 3.1. Mitophagy: Clearing Damaged Mitochondria

Mitophagy has emerged as a promising therapeutic target for SNHL, given its critical role in maintaining mitochondrial quality control and cellular homeostasis under stress conditions. This selective autophagy pathway, which specifically removes damaged or superfluous mitochondria via lysosomal degradation, serves as a vital mechanism to mitigate ototoxic damage [107,108]. The concept of mitophagy as a distinct autophagy subtype was established following the characterization of yeast mitochondrial degradation mechanisms in 2005. Since then, research has consistently shown that under ototoxic stress, dysfunctional mitochondria accumulate in cochlear cells, and impaired mitophagy exacerbates oxidative damage, thereby contributing to the pathogenesis of SNHL [109,110].

Mitochondrial clearance is vital due to mitochondria’s dual role in energy production and ROS generation. Through the TCA cycle and oxidative phosphorylation (OXPHOS), mitochondria produce ATP but also generate ROS as a byproduct. Dysfunctional mitochondria with impaired electron transport chain (ETC) function produce excessive ROS, causing oxidative stress that damages mitochondrial DNA (mtDNA) and ETC proteins, further reducing OXPHOS efficiency. This leads to mitochondrial membrane potential loss, triggering apoptosis and cochlear cell death [110]. Thus, timely removal of damaged organelles via mitophagy is crucial for auditory cell survival. Therapeutic targeting of mitophagy is supported by pharmacological studies. For instance, dynamin-related protein 1 (Drp1) inhibitor Mdivi-1 mitigates ototoxicity [111]. Similarly, metformin activates AMPK, triggers autophagy/mitophagy, prevents mitochondrial dysfunction, and suppresses apoptosis in TBHP-challenged HEI-OC1 cells [108]. Enhancing mitophagy reduces ototoxicity, though mitochondrial quality control regulation remains under active investigation [109,111].

**Figure 2:**
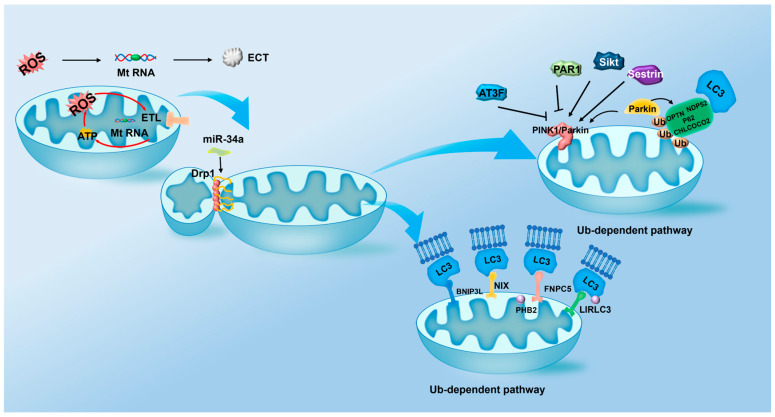
Dual-pathway model of mitophagy in cochlear hair cells. Damaged mitochondria are cleared via two parallel pathways: (1) Ubiquitin-dependent: Depolarization stabilizes PINK1, recruiting Parkin to ubiquitinate OMM proteins. Receptors (OPTN, NDP52) bind ubiquitin and LC3 to recruit mitochondria to autophagosomes. (2) Ubiquitin-independent: OMM receptors (BNIP3L/NIX, FUNDC1) and IMM receptor PHB2 directly bind LC3 via LIR motifs. Both pathways require Drp1-mediated fission and culminate in lysosomal degradation, crucial for preventing oxidative stress and ototoxicity.

Mitophagy involves coordinated signaling events: mitochondrial fission, labeling of damaged mitochondria via receptors, and encapsulation in autophagosomes for lysosomal degradation [112]. Its multi-step nature makes it vulnerable to dysregulation, compromising mitochondrial homeostasis. Evidence underscores mitophagy’s role in auditory dysfunction, necessitating detailed stage analysis in SNHL. We will delineate mitophagy mechanisms in SNHL by phase in subsequent sections (Figure 2).

#### 3.1.1. Mitochondrial Fission

The initial phase of mitophagy involves mitochondrial fission, serving as a crucial prerequisite. In mammalian cells, this fission process is driven primarily by Drp1. During fission, Drp1 mobilizes from the cytoplasm to the mitochondrial outer membrane, assembling into a helical complex that encircles the organelle. Mitochondria suffering dysfunction, characterized by depolarized membrane potential, subsequently undergo selective elimination via mitophagy [113,114]. Recognizing Drp1’s pivotal role in this pathway, Lin [109] established a cellular senescence model using C57BL/6 mouse HEI-OC1 cells and cochlear explants for early-onset hearing loss. This research revealed that inhibiting Drp1-dependent mitophagy triggers mitochondrial accumulation and disrupts ATP metabolism. Consequently, this dysfunction accelerates cochlear hair cell senescence and exacerbates hearing loss. Wang et al. further substantiated that suppressing miR-34a expression, thereby elevating Drp1 levels, enhances mitophagy and confers partial protection against cisplatin-induced ototoxicity [115]. Furthermore, the Gipc3 mutation potentially induces mitochondrial dysfunction by inhibiting a PH domain and leucine zipper motif 1 (APPL1)-mediated initiation of mitophagy. This inhibition likely curtails oxidative metabolism within HCs, representing the probable mechanism underlying SNHL caused by Gipc3 mutation [116]. These findings collectively establish mitochondrial fission as a critical and druggable regulatory node in auditory hair cell survival. Once fission is complete, the next critical step entails the selective tagging of damaged mitochondria through ubiquitin-dependent or independent receptors, a process ensuring precise target for degradation.

#### 3.1.2. Targeted Labeling by Independent Receptors

Following mitochondrial fission, the isolated organelles are selectively labeled for destruction through two principal pathways: the ubiquitin-dependent pathway and the ubiquitin-independent pathway. The ubiquitin-dependent pathway is primarily mediated by the PTEN-induced putative kinase 1 (PINK1)/Parkin axis.

PINK1 is a serine/threonine kinase essential for mitochondrial quality control, with a mitochondrial targeting signal and kinase domain, highly expressed in energy-demanding tissues [117]. As a mitochondrial integrity gatekeeper, it coordinates mitophagy [118]. Normally, PINK1 is imported into mitochondria, cleaved by PARL, and degraded [119,120]. In damaged mitochondria, PINK1 accumulates on the outer mitochondrial membrane (OMM), auto-phosphorylates, dimerizes, and recruits Parkin [121,122]. Parkin ubiquitinates OMM proteins, enabling receptors like OPTN and NDP52 to tether ubiquitinated mitochondria to autophagosomes via LIR domains for lysosomal degradation [122,123,124,125]. Evidence underscores the PINK1/Parkin pathway’s centrality in auditory protection. Yang et al. showed its activation protects against GM-induced ototoxicity by suppressing p53 signaling [126]. Xiong et al. found Sirt1 overexpression enhances PINK1/Parkin-mediated mitophagy, delaying age-related cochlear degeneration63. Pharmacological studies highlight therapeutic potential: Cho et al. reported Urolithin A (UA) activates PINK1/Parkin-dependent mitophagy, protecting cells from H_2_O_2_-induced senescence by downregulating p53 and p21, preserving mitochondrial function—effects abolished by Parkin knockdown [127]. Similarly, Parkin knockdown exacerbates cisplatin-induced mitochondrial dysfunction, underscoring Parkin’s protective role [128]. PINK1/Parkin effects are context-dependent and paradoxical: Sestrin 2 enhances ULK1/Parkin mitophagy to reduce noise damage, but reduced PINK1/Parkin expression protects against aminoglycoside toxicity [129]. This discrepancy may stem from a threshold effect: moderate stress (GM) activates protective mitophagy, while severe stress (neomycin) impairs the pathway via ATF3-mediated PINK1 repression, and inhibiting mitophagy may promote survival by avoiding excessive degradation [130]. Some studies report unchanged mitophagy with aminoglycosides, indicating mitophagy-independent ototoxicity mechanisms [2,131].

Following the ubiquitination of mitochondrial substrates by Parkin, cytoplasmic autophagy receptors are recruited to execute the final steps. Proteins such as p62/SQSTM1, OPTN, and NDP52 bind to the ubiquitin chains on the OMM and, via their LIR motifs, to LC3 on the phagophore, thereby bridging the damaged mitochondrion with the autophagic machinery. p62 itself is degraded along with the encapsulated cargo, making its protein level a negative correlate of autophagic flux—a classic autophagy marker whose alterations are frequently noted in ototoxicity studies [2,109,132]. Not all receptor recruitment is uniformly protective. Li et al. emphasize that excessive recruitment of Nuclear dot protein 52 (NDP52) can intensify neomycin-induced ototoxicity, highlighting the need for precise regulation of this process [133].

#### 3.1.3. Targeted Labeling by Ubiquitin-Dependent and Non-Ubiquitin-Dependent Pathways

Mitophagy can occur ubiquitin-independently via receptors that directly recruit autophagy machinery. Key receptors include BCL2/adenovirus E1B 19kDa interacting protein 3-like/Nip3-like protein X (BNIP3L/NIX), BNIP3, and FUN14 domain containing 1(FUNDC1) on OMM, and Pleckstrin homology domain-containing, family B, member 2 (PHB2) on the inner mitochondrial membrane (IMM), using LIR motifs to bind LC3 and bypass ubiquitination. BNIP3 and BNIP3L/NIX, homologous Bcl-2 proteins, are crucial for mitophagy initiation [134]. Aging research shows downregulation of BNIP3L/NIX and BNIP3 in cochlear tissues [135], reducing mitophagosome–lysosome colocalization and causing mitochondrial accumulation [135,136]. Ototoxicity models confirm decreased expression under H_2_O_2_ or cisplatin stress, indicating BNIP3L/NIX and BNIP3 protect cochlear cells by promoting mitophagic [127,128].

FUNDC1 is a key OMM receptor for ubiquitin-independent mitophagy, featuring an LIR motif that enables LC3 interaction [137]. Its phosphorylation status regulates mitophagy, with hypoxic stress promoting dephosphorylation to enhance LC3 binding and mitophagy stimulation. However, in ototoxic contexts like cisplatin exposure in HEI-OC1 cells, FUNDC1 expression remains stable, suggesting context-specific involvement [128]. When OMM rupture occurs, IMM protein PHB2 is exposed as a mitophagy receptor, interacting with the phagophore via its LIR motif [138]. Yu et al. demonstrated PHB2 expression in auditory cells and its mitophagy role [139]. Mitophagy and PHB2 decrease in ARHL mice but increase under H_2_O_2_-induced oxidative stress in HEI-OC1 cells, indicating PHB2’s critical role in ARHL pathogenesis [140].

Notably, as discussed in Section 2.4, the master regulator TFEB may globally coordinate mitophagy by ensuring sufficient lysosomal capacity to degrade engulfed mitochondria. Mitophagy is crucial for maintaining cochlear cellular homeostasis and reducing ototoxic damage. However, its complex regulation, involving multiple parallel pathways and receptors, requires further study.

### 3.2. Pexophagy: Mitigating Oxidative Stress

Pexophagy, a selective autophagy pathway, involves the degradation of peroxisomes into phagocytic vesicles in response to environmental stimuli [140]. Pexophagy has been reported to be associated with inflammation induced by LPS exposure, with impaired pexophagy leading to impaired peroxisome accumulation and reduction-oxidation (redox) imbalance [141]. Furthermore, pexophagy is implicated in noise-induced HCs damage. Specifically, overexposure to noise increases peroxisome levels in HCs and SGNs, and defective pexophagy contributes to NIHL [142]. Pejvakin, a peroxisome-associated protein belonging to the gasdermin family, plays a pivotal role in mitigating NIHL by preserving the normal three-dimensional ciliary ladder structure and enhancing mechanical transduction in HCs [143]. In response to noise exposure, Pejvakin can directly recruit LC3B to promote peroxisome selective autophagy (pexophagy), thereby protecting cochlear HCs from noise-induced damage [142,144]. Having explored the vital roles of selective autophagy pathways—specifically mitophagy and pexophagy—in safeguarding auditory cell viability under stress, we now synthesize these findings to chart future therapeutic directions and underscore the critical importance of autophagy regulation in SNHL.

## 4. Challenges and Translational Perspectives in Targeting Autophagy for SNHL Therapy

Translating autophagy-modulating therapies to the clinic faces multiple challenges: the blood-labyrinth barrier restricts drug delivery to the cochlea; the dual nature of autophagy requires precise, stage-specific intervention tailored to diverse SNHL etiologies; and chronic modulation of ubiquitously expressed regulators like mTOR raises safety concerns. A particularly critical hurdle is the lack of reliable non-invasive biomarkers to monitor autophagic flux in patients. Emerging liquid biopsy approaches offer promise in this regard: autophagy-related proteins (such as LC3-II and p62) or specific microRNAs detected in perilymph samples or blood-derived exosomes may reflect the autophagic status of the inner ear. Although still preclinical, near-infrared imaging of autophagy reporters in animal models suggests the feasibility of future non-invasive monitoring. Nevertheless, substantial challenges remain, including sensitivity, specificity, and the need to validate correlations with cochlear tissue autophagy. A critical consideration in translational research is the limitations of current preclinical models. For instance, the HEI-OC1 cell line, while useful for screening, may not fully replicate human hair cell physiology and drug responses, while mouse models differ in cochlear anatomy and pharmacokinetics. These interspecies differences highlight the need for complementary human-based models, such as iPSC-derived organoids, to validate findings prior to clinical application. Future advances in multi-omics and micro-sampling techniques may ultimately enable dynamic monitoring of autophagic molecules in perilymph, thereby guiding treatment decisions.

An additional consideration is how these emerging therapies might interact with current treatments for acute SNHL, such as corticosteroids, vitamin B12, and ATP. Corticosteroids may suppress autophagic flux, potentially counteracting autophagy-enhancing strategies, while ATP and vitamin B12 could support autophagy by improving mitochondrial function, offering possible synergistic effects. Thus, future autophagy-based interventions are unlikely to replace current therapies but rather may complement them—for example, combining acute anti-inflammatory action with stage-specific autophagy modulators for long-term hair cell survival. However, careful timing and patient stratification are essential to avoid unintended autophagy dysregulation, and well-designed clinical studies will be needed to determine optimal combination regimens.

Despite these challenges, autophagy remains a promising therapeutic target, with clinical strategies evolving along several fronts: pharmacological activation using compounds like UA and NAD+ precursors to enhance mitochondrial clearance, and indirect pathway engagement via repurposed drugs such as metformin. A particularly relevant context for otolaryngologists is the prevention of cisplatin-induced ototoxicity in patients undergoing chemoradiotherapy for head and neck malignancies. Several clinically available autophagy modulators—including rapamycin (mTOR inhibitor), metformin (AMPK activator), aspirin (FOXG1 activator), and certain statins (TFEB activators)—have shown otoprotective effects in preclinical studies. However, their repurposing for hearing preservation remains uncertain due to key considerations: potential interference with cisplatin’s antitumor efficacy, as autophagy modulation may affect cancer cell survival; the need for inner ear-specific delivery to avoid systemic immunosuppression or other off-target effects; and optimal timing relative to chemotherapy cycles. These concerns underscore the importance of developing targeted delivery systems—such as intratympanic injections, locally activated prodrugs, or nanoparticle formulations—that concentrate drugs in the cochlea while minimizing systemic exposure. For monogenic hearing loss, gene therapy offers potential to correct defective mitophagy genes directly. Ultimately, clinical application will depend on patient stratification and rigorous studies in head and neck cancer patients that balance otoprotection with oncologic outcomes. Until such data are available, routine use cannot be recommended, but these agents represent compelling avenues for future investigation. Although mTOR is a key therapeutic target, its wide expression and function in cellular processes raise concerns about long-term systemic manipulation. Continuous mTOR inhibition by Rapamycin causes immunosuppression and metabolic disorders, so localized delivery strategies are needed to concentrate therapeutic agents in the cochlea and reduce systemic exposure. Intratympanic injection of mTOR inhibitors, often combined with hydrogels for sustained release, shows potential in pre-clinical studies. Nanoparticle-based carriers and prodrug strategies with local enzymatic activation can improve specificity. Ultimately, the clinical viability of mTOR-targeted otoprotection depends on proving that localized delivery can be effective without the systemic toxicities of chronic oral administration.

## 5. Conclusions

In conclusion, autophagy’s central role in SNHL pathogenesis powerfully underscores its immense therapeutic promise. Importantly, any clinical application must carefully weigh the therapeutic benefits against potential systemic side effects, reinforcing the need for targeted delivery and personalized approaches. For established injury, a stage-specific approach is paramount: enhance protective autophagy early (such as via AMPK/SESN2 or pejvakin-LC3B pathways) to promote cell survival, but suppress excessive autophagy later to prevent cell death. Emerging targets like RIPOR2-GABARAP, mTOR-TFEB, and nanoparticles for targeted delivery illuminate promising next-generation otoprotection avenues.

A proactive preemptive strategy bolsters inner ear resilience by modulating key pathways for prevention, including: (1) Implementing preconditioning protection: Activate endogenous pathways (such as Brain-Derived Neurotrophic Factor (BDNF)) before insults like noise to reinforce autophagy, mitigating synaptic damage and abnormal flux. (2) Employing homeostatic enhancement: In a ARHL, leverage non-pharmacological approaches like exercise to sustain basal autophagy, clearing toxins and delaying presbycusis. (3) Leveraging circadian rhythm modulation: Regulate core clock genes to stabilize autophagy and counteract hearing damage from disruptions like sleep deprivation. To translate these insights into tangible therapies, critical methodological advances are essential. Single-cell multi-omics will delineate autophagy states across cochlear cells with unprecedented resolution, while deeper investigation clarifies the intricate interplay among mitophagy, pexophagy, and other selective autophagy pathways. Developing non-invasive autophagy biomarkers and sophisticated models like cochlear organoids is crucial for robust validation and translational progress. Combining precision medicine with systemic preemptive strategies opens multidimensional, low-risk, yet potent avenues for preserving auditory function across diverse SNHL manifestations.

## Figures and Tables

**Table 1:** Studies on different types of sensorineural hearing loss at different stages of autophagy.

Stage of Autophagy	Models	Targets	Autophagy	Effect	Ototoxicity	Reference
Autophagy initiation and phagophore formation	HEI-OC1 cells	miR-130b-3p↑, PPARγ, ATG5, Beclin-1↓	Decrease	Protection	ARHL	[34]
	HCs	Sestrin-2(SESN2)/AMPK/mTOR			GM-induced ototoxicity	[29]
		AKT↑, GSK-3β↓,	Increase		cisplatin-induced	[36]
	OSCs	SLC26A4 mutations	Increase			[17,42,43]
		OSBPL2 mutations	Decrease	protection		[44]
	SGNs	AMPK/ULK1	Increase	Protection	NIHL	[37]
	SGNs	PRDX1↑, PIP3↓, PTEN-AKT↓, mTOR↓	Increase	Protection	cisplatin-induced ototoxicity	[10,48,49]
	HEI-OC1 cells, HCs	YTHDF1↑, ATG14↑	Increase	Protection	cisplatin-induced ototoxicity	[50]
	HEI-OC1 cells	AMPK/FOXO3a↑, ATGs↑	Increase		cisplatin-induced ototoxicity	[52]
Formation of autophagosome	HCs	Sirtuin-1	Increase	Protection	ARHL	[62,63]
	HCs	Sirtuin-1	Increase	Protection	ARHL	[66]
	HEI-OC1 cells	NLRX1/ROS/JNK	Increase	Not protected	cisplatin-induced ototoxicity	[67,68]
	HEI-OC1 cells, C57BL/6J cochlear explants	RIPOR2, GABARAP	Increase	Not protected	aminoglycosides	[72]
	HCs	LPS, FOXG1↑, ROS↓	Increase	Protection	ARHL	[75]
	HCs	FOXG1↓, ROS↑	Decrease	Not protected	D-Gal induced aging rat model and a cellular model	[11]
	HEI-OC1 cells and CBA/CaJ mouse models	FOXG1↑, miR-34a↑, miR-96↑, miR-182↑, and miR-183↑	Increase	Protection	cisplatin-induced	[76]
Autolysosome formation	cochlear explant cultures and HEI-OC1 cells	UCHL1↓	Decrease	Not protected	GM-induced ototoxicity	[83]
	HEI-OC1 cells	Rab7↓	Decrease	Not protected	GM-induced ototoxicity	[23]
	HCs and HEI-OC1 cells	Dync1li1↓	Decrease	Not protected		[85]
Degradation of the contents	HEI-OC1 cells	TFEB↑, mTORC1↓	Increase	Protection	ARHL	[91]
		RONIN (THAP11), HCF1/HCFC1,TFEB	Increase	Protection	ARHL(D-Gal-induced hair cell aging)	[92]
	vascular margin cells in neonatal rats	HIF-1α, TFEB	Increase	Protection	ARHL	[93,94]
	HEI-OC1 cells, mouse cochlear explant culture	NaAsO2↓	Increase	Protection	APAP-induced auditory hair cell damage	[95]
	SGNs	TFEB↑, mTOR↓	Increase	Protection	degenerated SGNs	[101]
	HEI-OC1 cells and HCs within mouse cochlear explants	Atg5↑ and Atg7↑	Increase	Protection	acetaminophen (APAP) treated ototoxicity	[104]
	zebrafish	ATP6V1B1 mutations	Decrease	Not protected		[105]
	IHCs	SLC7A14	Increase	Not protected		[106]

↑ indicates upregulation of gene expression. ↓ indicates downregulation of gene expression.

## Data Availability

The original contributions presented in this study are included in the article. Further inquiries can be directed to the corresponding authors.

## Abbreviations

**Abbreviations**	**Full Forms**
SNHL	sensorineural hearing loss
ARHL	age-related hearing loss
mTOR	mammalian target of rapamycin
mTORC1	mammalian target of rapamycin complex 1
AMP	Adenosine monophosphate
AMPK	Adenosine monophosphate-activated protein kinase
ATG	autophagy-related gene
ULK1	unc-51 like autophagy activating kinase 1
ATG14	autophagy-related gene 14
PI3KC	phosphatidylinositol 3-kinase complex
HCs	hair cells
SGNs	spiral ganglion neurons
GM	gentamicin
HEI-OC1	House Ear Institute-Organ of Corti 1
SESN2	SESTRIN-2
GSK-3β	Glycogen synthase kinase-3β
AKT	protein kinase B
SLC26A4	Solute Carrier Family 26, Member 4
OSCs	outer sulcus cells
OSBPL2	Oxysterol Binding Protein-Like 2
PI3K	phosphatidylinositol 3-kinase
PIN1	Peptidyl Prolyl Cis-Trans Isomerase NIMA Interacting Protein 1
NIHL	noise-induced hearing loss
PRDX1	peroxiredoxin 1
H_2_O_2_	hydrogen peroxide
PTEN	phosphatase and tensin homolog deleted on chromosome ten
Vps34	Vacuolar protein sorting 34
Vps15	Vacuolar protein sorting-associated protein 15
YTHDF1	YT521-B Homology N6-Methyladenosine RNA Binding Protein
Ubl	ubiquitin-like
LC3	Microtubule-associated proteins light chain 3
GABARAP	Gamma-aminobutyric acid receptor-associated protein
ATG5	autophagy-related gene 5
SQSTM1	Sequestosome 1
ATG9	autophagy-related gene 9
NAD^+^	nicotinamide adenine dinucleotide
DNA	Deoxyribonucleic Acid
Sirt1	Sirtuin-1
ATG7	autophagy-related gene7
ATG8	autophagy-related gene 8
ATG9A	autophagy-related gene 9A
NLRX1	Nucleotide-binding domain and leucine-rich repeat-containing receptors family member X1
ROS	reactive oxygen species
JNK	c-Jun N-terminal kinase
ATG16L1	autophagy-related gene 16-like 1
RIPOR2	Rho-family interacting cell polarization regulator 2
FOX	Forkhead box
FOXO3a	Forkhead box O3a
BECN1	Beclin-1
BNIP3	BCL2 interacting protein 3
BNIP3L/NIX	BCL2/adenovirus E1B 19kDa interacting protein 3-like/Nip3-like protein X
PINK1	PTEN induced kinase 1
MAPK	mitogen-activated protein kinase
ATG4	autophagy-related gene 4 cysteine peptidase
FOXG1	Forkhead Box G1
LPS	lipopolysaccharide
UCHL1	Ubiquitin carboxyl-terminal hydrolase isozyme L1
Rab7	Ras-related protein Rab-7a
Dync1li1	Dynein, cytoplasmic 1, light intermediate chain 1
LC3B	Microtubule-associated protein 1 light chain 3 beta
TFEB	transcription Factor EB
MAPLC3B	Microtubule-associated protein 1 light chain 3 beta
SLC7A14	Solute carrier family 7 (cationic amino acid transporter, y^+^ system), member 14
DRP1	Dynamin-related protein 1
IMM	inner mitochondrial membrane
OMM	outer mitochondrial membrane
FUNDC1FU	N14 domain containing 1
LIR	LC3 interaction region
PHB2	Pleckstrin homology domain-containing, family B, member 2
UA	Urolithin A
NDP52	Nuclear dot protein 52
